# Program of active aging in a rural Mexican community: a qualitative approach

**DOI:** 10.1186/1471-2458-7-276

**Published:** 2007-10-03

**Authors:** María de la Luz Martínez-Maldonado, Elsa Correa-Muñoz, Víctor Manuel Mendoza-Núñez

**Affiliations:** 1 Universidad Nacional Autónoma de México, Unidad de Investigación en Gerontología (FES ZARAGOZA). Batalla 5 de mayo s/n, esq. Fuerte de Loreto, Col. Ejército de Oriente, 09230 México, D. F, México; 2 Doctorado en Salud Colectiva, Universidad Autónoma Metropolitana (Unidad Xochimilco), México D. F, México

## Abstract

**Background:**

Education is one of the key elements in the promotion of a thorough paradigm for active aging. The aim of this study is to analyze factors that contribute the empowerment of older adults in a rural Mexican community and, thus, promote active aging.

**Methods:**

The study was conducted in a rural Mexican community (Valle del Mezquital), based on an action-research paradigm. One hundred and fifty-five elderly subjects with elementary school education participated in a formal training program promoting gerontological development and health education. Participants in turn became coordinators of mutual-help groups (gerontological nucleus) in Mexico. In-depth interviews were carried out to assess the empowerment after training for active aging.

**Results:**

It was found that there was an increasing feeling of empowerment, creativity and self-fulfillment among participants. Among the main factors that positively influenced training of the elderly toward active aging were the teaching of gerontology topics themselves; besides, their motivation, the self-esteem, the increased undertaking of responsibility, the feeling of belonging to the group, and the sharing of information based on personal experience and on gerontological knowledge.

**Conclusion:**

The main factors that contribute to empowerment of older adults in a rural Mexican community for participate in active aging programs are the training and teaching of gerontology topics themselves; besides, their interest, experience and involvement.

## Background

The Political Declaration of the Second World Assembly on Aging states *"... the potential of older persons is a powerful basis for future development. This enables society to rely increasingly on the skills, experience and wisdom of older persons, not only to take the lead in their own betterment but also to participate actively in that of society as a whole" *[1].

The paradigm advanced by the World Health Organization defined active aging as *"the process of optimizing opportunities for health, participation and security in order to enhance the quality of life as people age" *[2].

Specifically, active aging refers to the empowerment of older persons in the biological, psychological and social areas, understanding empowerment as the individual's self-promotion, independence, and self-confidence, as well as his/her right to a dignified way of living according to self-imposed values, and the ability to stand for one's own rights, and to be free [3]. To become empowered, it is considered essential that the individual be kept informed about aging as a process, mainly within the perspective of community gerontology.

**Figure 1:**
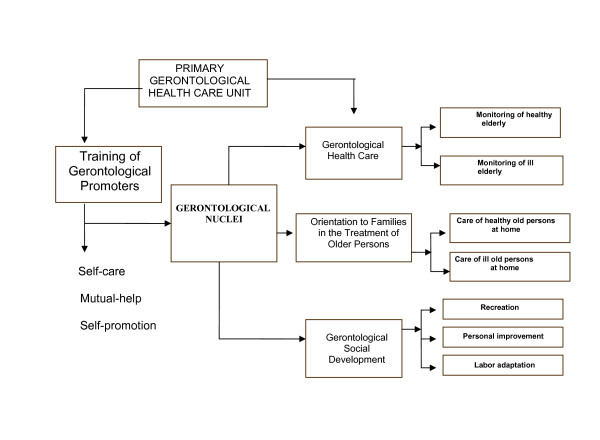
**Gerontological care model**. Primary Gerontological Health Care Unit is responsible for the design and implementation of educational programs and guides the training of the gerontological promoters for the development of mutual-help groups (gerontological nucleus). A gerontological nuclei is a group integrated by 10 to 15 older adults of nearby communities with similar interests. They are mainly involved in the practice of self-care, mutal-help, and self-promotion guidelines established by the program. The model is addressed toward the following objectives: (i) the supervision of the gerontological health status of participants in the program; (ii) the training of qualified gerontological health care promoters, certified on the basis of a formal continuous education program offered by a renowned academic institution; (iii) to provide orientation and guidance to families with regard to basic care practices with both healthy and sick older adults; (iv) to promote the social and gerontological development of the older populations in Mexico.

Our research group proposes a Gerontological Care Model framed with four key elements to achieve and promote an efficient level of empowerment in elderly populations (Figure 1). First, it is considered that knowledge is power, and providing education on gerontological, social, and health matters will provide the older adults with the tools they need to gain full access to the current health resources provided in Mexico, to fully promote their rights as citizens, and to promote the recognition of their needs and interests to government and nongovernmental agencies. Second, it is considered that inclusion and participation should be at the core of any educational initiative in the field. That is, the elderly population must be included at all stages of decision-making processes to guarantee both direct access to public and private health care, as well as a coherent response from their health and social institutions to their most demanding needs and interests. Third, it is believed that quality assurance and accountability by government officials, politicians, and those responsible for nongovernment organizations are of utmost importance. Therefore, it is necessary to establish horizontal mechanisms that guarantee the accurate evaluation of the performance, management and efficiency of those organizations by their ultimate beneficiaries: the elderly. Fourth, it is essential that local communities develop the capacity for both organization and team work to promote their autonomy and their direct participation in the negotiation of financial support for their community programs [4,5].

It is considered that self-care, mutual-help and the ability for self-negotiation constitute the fundamental strategic elements to be promoted and supported by the communitarian gerontological programs [6-9].

Education is one of the key elements in the promotion of a thorough paradigm for active aging. However, in some rural locations of Mexico a commonly held myth is that elderly have quite difficult to acquire new knowledge, which limit the implement of health promotion programs for this people group.

The critical approach to education for older adults has established the following four major principles [10-12]:

▪ *Mature individuals learn only what they want to learn*.

▪ *Mature individuals learn only what they are capable of learning*.

▪ *Mature individuals learn mainly what they teach themselves*.

▪ *Mature individuals learn mainly in terms of their experience*.

To summarize, the main purpose of this study is to analyze factors that contribute to empowerment of the older adults in a rural Mexican community to promote continuous and systematic active aging based on the theoretical and pedagogical principles previously outlined.

## Methods

The intervention program was based on an action-research paradigm. The study involved three main stages: (1) diagnostic evaluation; (2) intervention; and (3) follow-up. The evaluation of the intervention phase was made on the basis of questionnaires, recordings, documentary analysis, and field diaries. The main aspects of empowerment evaluated were the following:

### Personal dimension

Defined as the individual's ability to perform actions toward change and to develop self-confidence.

### Perceptions

Perceptions and stereotypes held by older persons with regard to their integration into the educational process.

### Knowledge

Level of previous education as well as specific knowledge acquired in the workshops and lectures offered as part of the program. Knowledge acquisition is considered as an important trait of empowerment to the extent that it allows older adults to improve their abilities to participate and to express their ideas, feelings, and opinions [4,13].

### Involvement

Level of involvement and participation as members of a group of promoters.

### Setting

The program was carried out in a rural area in Mexico (Valle del Mezquital, Hidalgo). It was based on a community health care model for the development of gerontological nucleus (Figure 1). This model was sustained in self-care, mutual-help and self-promotion defined as: (i) self-care: behaviors and actions with knowledge about aged-related biological changes, healthy life style, chronic disease prevention, social support net for elderly, carried out by the individual so as to take over general household and health promotion activities with the support of the family and the community; (ii) mutual-help: solidarity for social support, chronic disease prevention, instrumental-help when the partner is sick, and the social development of the gerontological nucleus; (iii) self-promotion: active social participation, for the best use of social-support nets for the elderly.

The gerontological nuclei were groups of mutual-help, formed by 20 older adults of nearby communities with similar interests, directed by Gerontological Promoters who discussed the topics of community gerontology with the group twice a week, and planned the actions of mutual-help and self-promotion, considering the needs of the group and social-support nets, besides the participation in the workshop. They formed thirty gerontological nuclei.

### Subjects

Adult male and female members of the community that met the following criteria: (a) interest in participating in an intensive training program focused on holistic gerontological development; (b) 60 to 74 years old; (c) literate; (d) absence of handicapping illnesses or serious visual or auditory disabilities; (e) leadership attributes and the ability to coordinate small groups. The subjects agreed to participate in the study after giving their informed consent. The Ethics Committee of the Universidad Nacional Autónoma de México (Zaragoza Campus) approved the research protocol for this study.

### Procedure

Screening and selection phase. Advertisements were distributed in the community specifying the objectives of the training program, admission criteria and commitments. Candidates were requested to submit a letter specifying the reasons underlying their interest to participate as well as any circumstance that could facilitate or affect their integration into the program.

### Intervention phase

It was centered on the implementation of a 60 hour workshop that integrated both theoretical and practical aspects (14 weekly sessions). An introductory text book entitled "Community Gerontology" was designed specifically for this project. It covered selected topics relevant to the type of activities and responsibilities expected from a gerontological promoter. It was printed taking into account its readability by older persons (font type and size). It also presented clear illustrations and diagrams related to everyday life situations as well as specific vignettes highlighting important ideas to facilitate meaningful learning [14]. A complementary workbook was designed including quizzes on all revised topics.

The topics of the workshop were selected and approved by a panel of four gerontologists, considering their basic knowledge on community gerontology, about age-related changes in the following aspects: biological, psychological and social, prevention of chronic diseases, healthy lifestyle in the aging period, empowerment, and social-support nets (Table 1), according to the paradigm for active aging.

**Table 1:** Workshop community gerontology topics

Age, aging and aged	Gender and aging	Nails care in the elderly
Second World Assembly on Aging	Sexuality in older adults	Foots care in the elderly
Active aging	Accidents in the elderly	Skin care in the elderly
Successful aging	Chronic diseases in the aging	Sleep hygiene in the elderly
Healthy aging and functionality	Prevention of diseases	Physical exercise and aging
Empowerment in the aging	Diabetes mellitus	Mouth and teeth care in the elderly
Gerontological promoters	Arterial hypertension	Dental prosthesis care in the aging
Self-care, mutual-help and self-promotion	Mild cognitive impairment	Social-support nets
Gerontological care model	Depression	Thanatology
Age-related biological changes	Cancer	Leisure and aging
Age-related psychological changes	Osteoporosis	Self-esteem and aging
Age-related social changes	Polypharmacy	Laws and aging
Ageism	Vaccination in the aging	Abuse and aging
The elderly and their families	Nutrition in the aging	Life quality and aging

The intervention included two phases, in the first phase simultaneously four workshops were started by four gerontologists for the study people (n = 155), which was divided in four groups, three of 40 persons and one of 35 participants.

In the second phase, the gerontologists selected thirty subjects between who had completed the first workshop for take the role of gerontological promoters (seven to eight of each group), considering their acquired knowledge, interest, and capacity for leadership, and who accepted the responsibility to drive a mutual-help group (gerontological nucleus). The selected subjects replicated their own training in a second workshop with others older subjects of the same community, planning actions of self-care, mutual-help and self-promotion.

The gerontological promoters had the function of imparting the acquired knowledge for extending their knowledge and actions about active aging. It was established that each promoter had to conduct at workshop in groups of 20 integrants, with the help of one participant from the initial workshop.

Some of the main pedagogical techniques employed during the workshop were: (1) lectures by specialists in the field; (2) presentations given by participants on specific topics; (3) group techniques addressed to retrieve information about particular life experiences; (4) intensive rehearsal and practice of skills; (5) group presentation and discussion of gerontological study cases.

### Follow-up phase

Included in the follow-up phase of the program, were supervision visits to the sites in the State of Hidalgo where the second workshop was imparted to the gerontological promoters.

### Evaluation

First, an analysis was made by members of the research team of video recordings of all sessions, considering the changes and evolution of behavior patterns in the older participants, as well as, their involvement and attitudes in both their training and their participation in community work. Second, in-depth interviews were carried out with participants showing the highest and lowest achievement levels in partial and final exams, as well as, with those participants who showed markedly different attitudes and involvement.

The empowerment was assessed, considering the acquired knowledge, healthy lifestyles adopted, and active participation in the group of mutual-help with actions of self-care and self-promotion.

### Data analysis

The quantitative data were analyzed using the SPSS 12.0 statistical program (SPSS Inc, Chicago, IL, USA). Descriptive statistics were percentages. Chi-square testing (Χ^2^) was used to compare proportions.

## Results

One hundred and fifty five subjects were selected to participate in the program (out of 205 applicants) for the workshop, 133 females (86%) and 22 males (14%). In this case, 100 older adults finished the program and 55 dropped out of the workshop, with a retention rate of 65% (Table 2). At the same time, 60 subjects who finished the first workshop were selected to act as gerentological promoters, who participated in the second workshop. Thirty groups of 20 older adults were formed, directed by two promoters for each one, to run the workshop, with the assistance of the four gerontologists. In the second workshop 600 persons participated and 500 finished the program, with a retention rate of 83% (Table 3). The difference of retention rate was statically significant in the second workshop rather than in the first workshop (65% vs. 83%, p < 0.001). At the same time, the principal reported causes for drop-out in the first workshop were: (i) Difficulty in apprenticeship 40%, (ii) domestic work 22%, (iii) grandchild care 18%, iv) diseases 10%, and v) unjustified causes 10%.

**Table 2:** Drop-out, and retention rates of first workshop

Groups	Initial participation	Drop-out	Finished the program	Retention rates (%)*
1	40	14	26	65
2	40	13	27	68
3	40	16	24	60
4	35	12	23	66
Total	155	55	100	65

*Retention rate = Finished program/Initial participation

**Table 3:** Drop-out, and retention rates of first workshop

Groups	Initial participation	Drop-out	Finished program	Retention rates (%)*
1	20	3	17	85
2	20	3	17	85
3	20	5	15	75
4	20	4	16	80
5	20	5	15	75
6	20	3	17	85
7	20	0	20	100
8	20	2	18	90
9	20	3	17	85
10	20	5	15	75
11	20	4	16	80
12	20	5	15	75
13	20	7	13	65
14	20	5	15	75
15	20	3	17	85
16	20	1	19	95
17	20	3	17	85
18	20	4	16	80
19	20	5	15	75
20	20	0	20	100
21	20	6	14	70
22	20	7	13	65
23	20	1	19	95
24	20	2	18	90
25	20	3	17	85
26	20	5	15	75
27	20	0	20	100
28	20	2	18	90
29	20	3	17	85
30	20	1	19	95
Total	600	100	500	83

*Retention rate = Finished program/Initial participation

In the second workshop the causes for drop-out were: (i) difficulty in apprenticeship 26%, (ii) domestic work 38%, (iii) grandchild care 27%, (iv) diseases 5%, (v) unjustified causes 4%.

As for the academic achievement, all participants complied with the originally established evaluation criteria. That is, they submitted homework papers, handed in the completed quizzes in the workbook, passed course examinations satisfactorily, presented and participated in the analysis of a case study, carried out supervised practice sessions, and provided preliminary information about the new gerontological group they had started, so as to apply and replicate the information received during training (Table 4).

**Table 4:** The educational process for active aging: Qualitative analysis

**Indicator**	**Analysis**
***Mature individuals learn only what they want to learn***.	The interest in learning is permeated by the personal life store of the older person as well as by interests and motivations of the individual at a given moment in their lives: "*I always wanted to become a health promoter, now I have been able to reach that dream*."
	Participants were selective with regard to the knowledge they received as a function of their expectancies, motives and intentions. For some of them practical matters represented their main learning objectives whereas for others theoretical contents were more readily appropriated.
***Mature individuals learn only what they are capable of learning***.	Previous education was a strong determinant of academic achievement. Hence, those participants with primary school education showed more difficulties both in the integration of theoretical contents and in answering exams. Those with access to higher education were more active during the teaching-learning process: *"I taught mathematics in secondary school, I have always been restless and I like to keep on learning"*.
***Mature individuals learn mainly what they teach themselves***.	Older persons assume the responsibility of their own learning. It was found that the best pedagogical approach with older persons was to promote self-learning as a way to appropriate knowledge. *"I have been doing my homework ... I have gone back to my book as many times as necessary ... sometimes I forget things but I check in my book"*.
*Mature individuals learn mainly in terms of their experience*.	Older individuals hold deeply rooted cultural perceptions of learning. In this sample was observed a tendency towards passiveness and defensiveness. However, the use of instructional strategies focused on the enforcement of participation and on the discussion of individual experiences proved extremely helpful to overcome those reactions. "*I want to speak about my own experience. Your pedagogical experience *[that of the researchers] *was crucial for meeting our educational objectives ... thanks for being with us ..."*.

### The personal dimension

The personal dimension of empowerment, it was found that some of the older persons who participated in the educational process began to perform actions toward change. That is, they developed a sense of self-confidence, which motivated them to carry out suggested actions, even when they had originally been hesitant to do so, on account of self-perceptions related to limited knowledge and an inability to address the group for fear of not being listened to by others. Participants with and without previous experience began to invite other older adults in their communities to get involved in the program, to share with them the information acquired, and to carry out actions that had never been carried out before. Furthermore, the way in which they started to organize themselves to start integrating groups to conduct those activities, and becoming aware of the potential of each individual to best achieve the group objectives is a empiric element of empowerment. A different division of responsibilities became evident, in which, those with better communication skills assumed the roles of instructors whereas, those strong in practical skills got involved in organizational matters. In addition, some of the female participants commented on the importance of the information given as part of the educational program and the way it had impacted their personal lives (e.g., some had already taken the initiative to undergo a medical examination). Reports of female participants underscored the importance of achieving a personal knowledge of their body changes as a result of the aging process.

The ability to participate and to promote changes in their immediate environment was evident at two main levels: the family and the community. In the first case, participant remarks included the following:

▪ *"At home they ask me what I am doing, they tell me that I look different, they value the teachings I bring home, they say that I am doing things that I had never done before."*

▪ *"Now my cooking is different, I try not to use so much fat and I am eating more fruit and vegetables, I think this will be good for my grandchildren."*

### Perceptions

With regard to their ability to bring about changes in their own communities, illustrative excerpts are as follows:

▪ *"It is very important for us to take what we have learned here to those places where there are greater needs, where older adults cannot participate because of the distance of their homes, because of their lack of opportunities, or because no one pays attention to them."*

▪ *"We can now express our ideas and be listened to."*

▪ *"For us it has been very gratifying to be able to tell other older adults how important they are, to be able to share our time with them, and to share some of the knowledge and experience that we have acquired in the program."*

### Knowledge

It was also observed that the process of empowerment at those levels was different for each participant. That is, although for some of them the process of empowerment tended to be rather fast and immediate, it was more gradual and slower for others, making evident the influence of factors, such as, personal history, previous education, working experience, as well as, the roles they had previously played as social agents.

In addition, the qualitative analysis of information gathered through field diaries, interviews, recordings, and videos taken throughout the program showed that the participants gradually internalized and appropriated both the knowledge and the philosophy underlying the models of gerontological nuclei, as is evident in the following excerpts of our elderly promoters in the final evaluation session:

▪ *"We cannot work, or pretend to do anything, if it not rooted in self-care, self-help and self-promotion"*.

▪ *"We have to analyze first the situation of the elder person, get information about his/her interests and health status and provide him/her with guidance and social assistance"*.

▪ *"We, as mature adults, still have very many things to do. This workshop has opened for us the possibility to share all this knowledge with others"*.

The gerontological promoters at the beginning of the formative phase, considered the main limitation affecting the possible outcome of the program as "the difficulty to learn". Among other things, they mentioned the following concerns:

▪ *"I am afraid of not being able to learn"; "I cannot memorize things as I used to"; "It's been a long time since I last studied"; "We are too old and we forget things"*.

### Involvement

Field diaries gave evidence of how the older adults gradually adapted to the program, increasing systematically their participation and involvement, gaining confidence during exams, and interacting more and more with their peers. In this regard, it is important to note that the group soon became a highly important source of social support, most of them being very punctual and persevering.

The self-care dimension, promoters carried out actions directly addressed toward the prevention and early detection of particular diseases. As a standard practice during all their meetings, pulse pressure and body weight were measured. They also established contact with health care agencies and social organization in their communities, taking positive actions such as negotiating mastographia and Papanicolaou tests for all female members of their groups, systematic blood testing, reduction in prescription glasses, as well as free-outpatient consultations in community hospitals. A further objective at this stage was to reach communities and groups with a higher demand for those services including remote rural areas, social rehabilitation centers, self-help groups, nongovernmental organizations, and so on. Examples of the older adults' achievements and perspectives can be illustrated in the following excerpts:

▪ *"Laura is the one who gives the talks. To make them understand us better, we always refer back to the history of Ixmiquilpan *[their home town]. *What it used to be and why we are facing certain types of illness now."*

▪ *"We try to get to know everyone. We want to know who they are and what they do."*

▪ *"We have tried to make use of social support agencies. The owner of a bathing resort in our community has already agreed not to charge admission fees for older persons."*

▪ "We are not just giving them information. We also try to socialize with them and share our food with them".

Lastly, as to the development of attitudes and aptitudes for leadership, it was found that participants with a previous experience in the conduction of groups (e.g., retired teachers) were the ones that showed a greater disposition to become coordinators of gerontological nuclei, although the others were increasingly exploring that facet of their personalities.

## Discussion

Informal help has been acknowledged as a necessity for healthy and frail older people in community care [15-18]. During the Second World Assembly on Aging, the need to develop community health care models under an active aging paradigm was pointed out [1]. For this reason, it is a conviction that training older adults as promoters of gerontological health programs is a major undertaking in such a direction.

The development of a feeling of empowerment through the implementation of educational intervention programs focused on active aging, constitutes a key element to guarantee successful community intervention that may eventually have a positive impact on the health and quality of life of the older population [19,20].

The results here made evident a systematic and gradual process of empowerment in the participants of program, with their assuming an active and leading role within their communities, developing specific abilities to promote actions toward change, and increasing their feelings of self-confidence. It is important to emphasize that the imparting of relevant knowledge played a fundamental role in moving toward such an objective.

However, even when it can be said that most of the key objectives of the intervention program were met, it has to be acknowledged that empowerment was not as generalized as originally expected, because this process is dependent on individual variables, becoming much slower and limited in certain subjects. Specifically, not all of those levels of empowerment outlined by Roelands have been achieved by all participants [4], but in the case of those who have made evident a rapid movement toward that goal it is a certainty certain that it has had an impact on their personal lives and in their interaction within their communities, thus paving the way toward active aging. In this study, women had greater participation than man in actions of self-negotiation and adoption of healthy life styles. This could be due the women of several rural communities of Mexico have a roll linked to care of the family, and therefore they have more interaction with social and health institutions than the man. However, in others studies has been found that the voices of older women are rarely heard in debates about the health of the disadvantaged groups and that their potential to positively contribute to family and community health care is seldom acknowledged [21,22].

Our results show that the active aging paradigm constitutes a feasible option to face current challenges related to aging in developing countries. Five months to finish the second workshop, five hundred older adults are participating in the program with actions of active aging in neighboring towns and communities. Therefore is an error considerer to older adult population as "objects", when the reality shows that they are "active subjects".

These results demonstrate that when the older persons are given the necessary tools, they can develop successful and creative coping strategies that go well beyond original expectancies. In this sense, it is important to outline that the retention rate was significantly higher (p < 0.001) in the second workshop, when the knowledge was imparted by the gerontological promoters, probably because of the language used, information framed in the context, communication (elderly-elderly), and a relaxed environmental.

Moreover, training older adults as promoters for active aging provides them with an opportunity to construct spaces of social and emotional change, and interact within the particular social contexts in which they are immersed, increasing feeling of belonging and identity.

Finally the empowerment process during this intervention program was not linear, without it was varied according to the participants' life experiences, to their personal histories, and to their level of subordination within the family sphere.

## Conclusion

Our results showed that the main factors that contribute to empowerment of older adults in a rural Mexican community for participate in active aging programs are the training and teaching of gerontology topics themselves; besides, their interest, experience and involvement. Therefore the empowerment in older adults to reach active aging is feasible with training programs that considering the participation themselves in the teaching of gerontology topics.

## Competing interests

The author(s) declare that they have no competing interests.

## Authors' contributions

MLM participated in the design of the study, participated in the subject interviews and helped to draft the manuscript. EC participated in the subject interviews and helped to draft the manuscript. VMM conceived and designed the study, developed the interview and survey questions, recruited participants, participated in the subject interviews, coded transcripts, and drafted the manuscript. All authors read and approved the final manuscript.

## Pre-publication history

The pre-publication history for this paper can be accessed here:


